# Optimization of Irrigation Amount and Nitrogen Rate of Drip-Fertigated Sugar Beet Based on Sugar Yield, Nitrogen Use Efficiency, and Critical Nitrogen Dilution Curve in the Arid Southern Xinjiang of China

**DOI:** 10.3390/plants14132055

**Published:** 2025-07-04

**Authors:** Ying Wang, Fulai Yan, Junliang Fan, Fucang Zhang

**Affiliations:** Key Laboratory of Agricultural Soil and Water Engineering in Arid and Semiarid Areas of Ministry of Education, Northwest A&F University, Yangling 712100, China; wangying930313@163.com (Y.W.); nwwfjl@163.com (J.F.)

**Keywords:** sugar yield, nitrogen (N) use efficiency, N concentration, N nutrition index, soil nitrate nitrogen

## Abstract

The critical nitrogen (N) dilution curve is widely used to diagnose crop N status, but no such model has been developed for sugar beet. This study evaluated the effects of irrigation amount and N rate on sugar yield, N use efficiency, and soil nitrate-N (NO_3_-N) residue of drip-fertigated sugar beet in the arid southern Xinjiang of China. A reliable N nutrition index (NNI) for sugar yield was also established based on a critical N dilution curve derived from the dry matter of sugar beet. A three-year field experiment was established with six N rates (25–480 kg N ha^−1^) and three irrigation levels based on crop evapotranspiration (*ET_c_*) (0.6, 0.8, and 1.0 *ET_c_* in 2019 and 2020, and 0.4, 0.6, and 0.8 *ET_c_* in 2021). Results showed that sugar yield and N uptake increased and then generally stabilized with increasing N rate, while N use efficiency decreased. Most soil NO_3_-N was mainly distributed in the 0–60 cm soil layer, but increasing irrigation amount reduced residual NO_3_-N in the 0–80 cm soil layer. Additionally, the established critical N dilution curve of sugar beet was considered stable (Normalized RMSE = 16.6%), and can be used to calculate plant N requirements and further N rates during sugar beet growth. The results indicated that the optimal NNI was 0.97 under 0.6 *ET_c_* for sugar yield production of sugar beet in this study. This study provides a basis for efficient water and N management in sugar beet production in arid and semi-arid regions globally.

## 1. Introduction

Irrigation and nitrogen (N) application are the two most important agricultural practices for improving crop growth and yield in arid and semi-arid areas [1,2,3]. In modern agricultural production, it is common that people apply excessive irrigation and N fertilizer to maximize crop yields [4,5]. Recently, fertigation using partially treated wastewater has also been explored as a way to recycle water and nutrients in arid regions [6]. However, these agricultural practices ignore the synchronization between plant N demand and supply [7,8]. Many researchers have reported that this practice not only reduces crop yield and N use efficiency but also deteriorates the soil environment and poses a serious threat to the sustainable development of agriculture [9,10,11]. However, insufficient irrigation and N supply also limit plant growth and decrease crop yields [12]. Therefore, it is crucial to determine the required amount of irrigation and N fertilization based on the estimated water and N requirements of a specific crop [13,14].

Sugar beet (*Beta vulgaris* L.) is a common industrial crop that is salt-tolerant [15] and has a high economic benefit for farmers. Drip irrigation under plastic mulch is widely used for agricultural production in arid and semi-arid regions because this technology can alleviate the shortage of agricultural water resources and stimulate the sustainable production of local crops. For this reason, sugar beet has been widely cultivated in Xinjiang, Northwest China. Sugar beet has high water requirements, and the sugar yield of sugar beet is greatly affected by the N rate [16,17]. Thus, ensuring a balanced supply of water and N fertilizer can lead to high sugar yields [18,19]. However, people in the regions blindly apply irrigation water and N fertilizer, resulting in stunted growth of sugar beet [20], a decline in yield, water and fertilizer use efficiencies, and a waste of water and fertilizer resources [12,21], with the attendant environmental problems. The amount of N applied to sugar beet depends on factors such as the demand for N in the forms of nitrate (NO_3_^−^) and ammonium (NH_4_^+^) for crop growth, as well as the availability of these N forms in the soil [7,22], which indicates that the optimal N rate required in sugar beet production is uncertain [8]. Early on, Bu et al. [23,24] used remote sensing technology to monitor N concentration as a guide to predict the yield of sugar beet. However, it is difficult to apply this technology in most areas due to its high cost.

In previous studies, there has been a lot of focus on the accurate diagnosis of plant N concentration, including the critical N (*N_c_*) dilution curve of crops and the N nutrition index (NNI) [25,26,27]. The *N_c_* dilution curve and NNI are usually used to evaluate crop N requirements for reliable N recommendations [28]. The *N_c_* dilution curve can be defined as the minimum N concentration at the maximum biomass during a certain growth period, which is usually based on the dry matter of stems and leaves or the whole plant [29]. Calculating the NNI can quantify crop N status, which is a reliable indicator for evaluating crop N nutrition status [30]. Previous studies have reported different *N_c_* dilution curve models, including general ones and specific ones for C3 or C4 crops [31]. However, there are differences in the N uptake dynamics of different crops, and there is also an effect of climate. When a model is applied to a new climate region and crops, the model parameters need to be calibrated. Therefore, each crop should have its own *N_c_* dilution curve model, considering the characteristics of the crop and the prevailing meteorological conditions [32,33].

Many studies have established the *N_c_* dilution curve models based on aboveground biomass for different crops, including maize [34,35], rice [36], potato [37], and tomato [38]. The dry matter of sugar beet is readily available, and an *N_c_* dilution curve model [28] can be used to calculate the current N concentration of the plant, which can be used to recommend the optimal N fertilization requirements. To address this, a three-year field experiment was conducted on sugar beet to establish an *N_c_* dilution curve model of sugar beet under drip irrigation with plastic mulch for guiding sugar beet production in arid and semi-arid areas. Therefore, the objective of this study was to investigate the effects of irrigation level and N rate on sugar yield, N use efficiency, and soil nutrient residue, and to develop an Nc dilution curve model for sugar beet under drip irrigation in arid and semi-arid regions to support site-specific N management.

## 2. Materials and Methods

### 2.1. Experimental Site Description

A three-year field experiment was carried out on sugar beet in 2019, 2020, and 2021 at the test station of Thirty-One Group in Korla (40°53′ N, 86°56′ E; 900 m elevation), Xinjiang Province, Northwest China [39]. The experimental site is located in the continental desert climate region with an annual average precipitation of 59 mm and an annual mean temperature of 11 °C. The soil texture is Alfisol, which is sandy loam-textured with 2% clay, 38% silt, and 60% sand in the 0–80 cm soil layer, and the pH is 8.7. The bulk density is 1.61 g cm^−3^ and the water content at field capacity is 0.18 cm^3^ cm^−3^. The soil is slightly salinized with an average soil salt content of 1.07 g kg^−1^. The experimental site had soil NO_3_^−^-N of 13.79 mg kg^−1^, available P of 6.52 mg kg^−1,^ and available K of 25.32 mg kg^−1^. Details about meteorological data at the study site during the three years can be found in Figure S1.

### 2.2. Experimental Design

The N treatments consisted of 25, 60, 120, 240, 360, and 480 kg N ha^−1^ in the three years (designed as N_25_, N_60_, N_120_, N_240_, N_360_, and N_480_, respectively). The phosphorus and potassium rates were 120 kg P_2_O_5_ ha^−1^ and 60 kg K_2_O ha^−1^, respectively. Urea (46% N), ammonium dihydrogen phosphate (11% N; 53% P_2_O_5_), and potassium oxide (60% K_2_O) were used as fertilizers. The irrigation amounts were based on fractions of crop evapotranspiration (*ET_c_*) and combined with three irrigation coefficients (0.60, 0.80, and 1.00) in 2019 and 2020, which were denoted as *W*_0.6_ (0.60 *ET_c_*), *W*_0.8_ (0.80 *ET_c_*), and *W*_1.0_ (1.00 *ET_c_*), respectively. However, it was found that there was no significant difference in taproot yield between *W*_0.6_ and *W*_0.8_ in 2019 and 2020, and root yield showed a downward trend under *W*_1.0_. Therefore, the three irrigation coefficients were adjusted to 0.40, 0.60, and 0.80, so the three irrigation amounts became *W_0_*_.4_ (0.40 *ET_c_*), *W*_0.6_ (0.60 *ET_c_*), and *W*_0.8_ (0.80 *ET_c_*) in 2021, respectively. *ET_c_* was calculated as follows [40]:

(1) $${ET}_{c}={\;K}_{c}\;\times \;{ET}_{0}$$

where *ET*_0_ is the reference crop evapotranspiration calculated according to the FAO-56 Penman–Monteith equation during the sugar beet growing seasons of 2019, 2020, and 2021 (Figure S1); *K_c_* is the crop coefficient for sugar beet, which was determined according to the sugar beet growth stage. The *K_c_* values used were 0.32 at the early stage, 0.97 at the middle stage, and 0.77 at the late stage [41]. *ET*_0_ was calculated as follows [40]:

(2) $${ET}_{0}=\frac{0.408\Delta \left(R_{n}-G\right)+\gamma \frac{900}{T_{mean}+273}u_{2}(e_{s}-e_{a})}{\Delta +\gamma (1+0.34u_{2})}$$

where ${ET}_{0}$ is the reference evapotranspiration (mm·day^−1^), Δ is the slope of the saturation vapor pressure curve at air temperature (kPa·C^−1^), $R_{n}$ is the net radiation at the crop surface (MJ·m^−2^·d^−1^), G is the soil heat flux density (MJ·m^−2^·d^−1^), γ is the psychometric constant = (0.665 × 10^−3^ × P), kPa·C^−1^ [40], $u_{2}$ is the wind speed at 2 m height (m·s^−1^), $e_{s}$ is the saturation vapor pressure (kPa), $e_{a}$ is the actual vapor pressure (kPa); ($e_{s}-e_{a}$) is the saturation vapor pressure deficit (kPa), and $T_{mean}$ is the mean daily air temperature at 2 m height (°C).

Each treatment was set up in triplicate field plots and a randomized complete factorial block design (4.6 m wide and 12 m long). The sugar beet variety “*BETA356*” was planted on 12 April 2019, 13 April 2020, and 12 April 2021, respectively. The drip irrigation and fertilization system under plastic mulch included a water pump, filter, plastic film, fertilization tanks, and water distribution pipelines. The drip tape was an in-line drip tape with drippers spaced 30 cm apart. The dripper flow rate was 2.4 L h^−1^ and the dripper operating pressure was 0.1 MPa. The experimental area adopted the drip irrigation mode of one film, two belt lines with three beet lines, that is, two drip irrigation belts were laid under one film, and three lines of beets were planted. The width of the film was 106 cm and the width of the two bare areas between the films was 46 cm. Two drip irrigation belts were laid under the film at a distance of 30 cm from the edge of the film, and three rows of sugar beet were planted at a distance of 20 cm from the edge of the film and between the two drip irrigation belts, respectively. The planting spacing of sugar beet was 30 cm (Figure S2).

There were six fertilization events during the whole growth period (Figure S3). Fertilizers were applied before the middle stage of the sugar beet. The irrigation dates of each treatment were the same, and the irrigation interval was 7 days (Figure S3). The irrigation began in early June and stopped in late August. During sugar beet growing seasons, the actual irrigation amounts were 136 mm under *W*_0.4_ in 2021; 186 mm, 198 mm, and 197 mm under *W*_0.6_ in 2019, 2020, and 2021; 244 mm, 260 mm, and 258 mm under *W*_0.8_ in 2019, 2020, and 2021; and 301 mm and 322 mm under *W*_1.0_ in 2019 and 2020, respectively (Figure S3).

### 2.3. Measurements and Methods

#### 2.3.1. Sample Analyses

##### Sugar Yield

All sugar beets of each plot were harvested, the leaves were removed, and the fresh taproots were washed, cleaned, and weighed to determine sugar beet taproot yield per plant and taproot yield per unit area. After calculating the taproot yield, three representative fresh taproots from each plot were further used to measure sucrose content, with a total of nine fresh taproots for each treatment. Sucrose content was measured with a saccharometer measure (PAL-1, Tokyo, Japan) (%) and the sugar yield per plant was determined. The sugar yield was further calculated as follows [42]:

Sugar yield (t ha^−1^) = taproot yield (t ha^−1^) × sucrose content (%) (3)

According to Ebmeyer and Hoffmann [43], relative sugar yield is the sugar yield in relation to the grand mean of all N treatments in 2019, 2020 and 2021.

##### N Concentration and Uptake

Three representative plants were sampled to determine the total dry matter on 67, 89, 111, 137, and 161 days after planting in 2019, 78, 98, 118, and 160 days after planting in 2020, and 81, 101, 120, 140, and 162 days after planting in 2021 each plot, respectively. Fresh samples were immediately dried in an oven at 105 °C for 30 min for initial drying and subsequently dried at 75 °C to constant weight. After calculating the total dry matter, the samples were ground, extracted with H_2_SO_4_-H_2_O_2_, and analyzed for N concentration (g kg^−1^) with a continuous-flow auto-analyzer. The total plant N uptake was further calculated as follows:

Sugar yield (t ha^−1^) = taproot yield (t ha^−1^) × sucrose content (%) (4)

##### N Use Efficiency

According to Ebmeyer and Hoffmann [43], N uptake efficiency (NU_P_E), N utilization efficiency (NU_t_E), and N use efficiency (NUE) of sugar beet were calculated as follows:

(5) $${\mathrm{NU}}_{p}E=\frac{N\;\mathrm{uptake}\;(\mathrm{kg}\;{\mathrm{ha}}^{-1})}{N\;\mathrm{rate}\;(\mathrm{kg}\;{\mathrm{ha}}^{-1})}$$

(6) $${\mathrm{NU}}_{t}E=\frac{\mathrm{Sugar}\;\mathrm{yield}\;(\mathrm{kg}\;{\mathrm{ha}}^{-1})}{N\;\mathrm{uptake}\;(\mathrm{kg}\;{\mathrm{ha}}^{-1})}$$

(7) $$\mathrm{NUE}=\frac{\mathrm{Sugar}\;\mathrm{yield}\;(\mathrm{kg}\;{\mathrm{ha}}^{-1})}{N\;\mathrm{rate}\;(\mathrm{kg}\;{\mathrm{ha}}^{-1})}$$

##### Soil NO_3_-N Residue

Ammonium nitrogen (NH_4_-N) is easily absorbed by crops and not easily leached in the deep soil [44], so we have mainly focused on the content of nitrate–nitrogen (NO_3_-N) in the soil. After the sugar beet was harvested, soil samples were taken by the soil drilling method. Samples at four horizontal locations (0, 20, 30, and 53cm from the edge of the plastic film in the 0–20 cm, 20–40cm, 40–60 cm, and 60–80 cm soil layers were collected) (Figure S2). The soil samples were subjected to air dry and fine screening, 2 mol L^−1^ Potassium Chloride (*KCl*) solution was then used for extraction (dry soil 5 g, soil liquid ratio 1:10), and the content of NO_3_-N in the soil was determined by flow analyzer.

#### 2.3.2. Critical Nitrogen (N_c_) Dilution Curve

##### N_c_ Dilution Curve Based on Dry Matter

The *N_c_* dilution curve model was used to evaluate the N nutrition status of sugar beet. The *N_c_* dilution curve was calculated as follows [28]:

(8) $$N_{c}=a\;{\mathrm{DM}}_{\max}^{-b}$$

where $N_{c}$ is the *N_c_* concentration (g kg^−1^); a is the N concentration for DM = 1 Mg ha^−1^ [32]; ${DM}_{max}$ is the highest dry matter at the minimum N rate (Figure S4, Mg ha^−1^); b is dimensionless and represents the ratio between the relative decline in plant N concentration and the relative crop growth rate [45]. The dry matter and N concentration samples in 2020 and 2021 (*n* = 5, 9, 9, and 4 under *W*_0.4_, *W*_0.6_, *W*_0.8_, and *W*_1.0_, respectively) were used for model development since they included all irrigation treatments, and the dry matter and N concentration in 2019 (*n* = 5, 5 and 5 under *W*_0.6_, *W*_0.8_, and *W*_1.0_, respectively) was used for model validation (Figure S4).

##### Evaluation of Model Performance

Root Mean Square Error (RMSE) and Normalized Root Mean Square Error (n-RMSE) were used to validate the model, and a 1:1 scatter plot between the simulated *N_c_* value and the measured value was used to ascertain the reliability of the model. The RMSE and n-RMSE were calculated as [1,29]:

(9) $$\mathrm{RMSE}=\sqrt{\frac{\sum_{i=1}^{n}{\left(S_{i}-M_{i}\right)}^{2}}{n}}$$

(10) $$n-\mathrm{RMSE}=\frac{\mathrm{RMSE}}{\bar{m}}\;\times \;100\%$$

where $S_{i}$ is the simulated value; $M_{i}$ is the measured value; n is the data number; $\bar{m}$ is the average of observed values. Generally, with n-RMSE < 10%, a model is considered to be extremely stable; 10% < n-RMSE < 20% is considered stable; 20% < n-RMSE < 30% is considered generally stable; n-RMSE > 30% is considered to have poor stability.

##### Nitrogen Nutrition Index

The N nutrition index (NNI) is an important indicator to determine the N nutritional status of crops [46,47]. The NNI was calculated as follows [48]:

(11) $$\mathrm{NNI}=\frac{N_{i}}{N_{c}}$$

where $N_{i}$ is the actual N concentration (%). Values of NNI < 1 indicate that biomass growth was limited by the N supply, while a value of NNI = 1 indicates that biomass growth was not limited by the N supply, and values of NNI > 1 indicate crops under luxury N conditions.

### 2.4. Data Analysis and Statistics

Data were compiled and analyzed using Excel 2010 (Microsoft Corporation, Albuquerque, NM, USA). SPSS 24.0 was used for statistical analyses (SPSS Inc. Chicago, IL, USA). Origin 2018 was used to create the figures. Before statistical analysis, we first assessed the normality (Shapiro–Wilk test) and constant variance (computing the Spearman rank correlation between the absolute values of the residuals and the observed values of HA) of the data. The two-way (irrigation by N) analysis of variance (ANOVA) was used to assess the treatment effects on sugar yield, N uptake, NU_P_E, NU_t_E and NUE in each experimental year. In order to calculate the *N_c_* concentration values, the dry matter values in different N rates under each irrigation amount in each experimental year were compared using one-way ANOVA (SPSS Inc. Chicago, IL, USA) by Tukey’s HSD test at the 5% level (*p* < 0.05). All pairwise comparisons of the treatment means were performed using ANOVA (SPSS Inc. Chicago, IL, USA) by Tukey’s HSD test at the 5% level (*p* < 0.05).

## 3. Results

### 3.1. Sugar Yield, N Uptake, Use Efficiency, and Residual

#### 3.1.1. Sugar Yield

**Figure 1:**
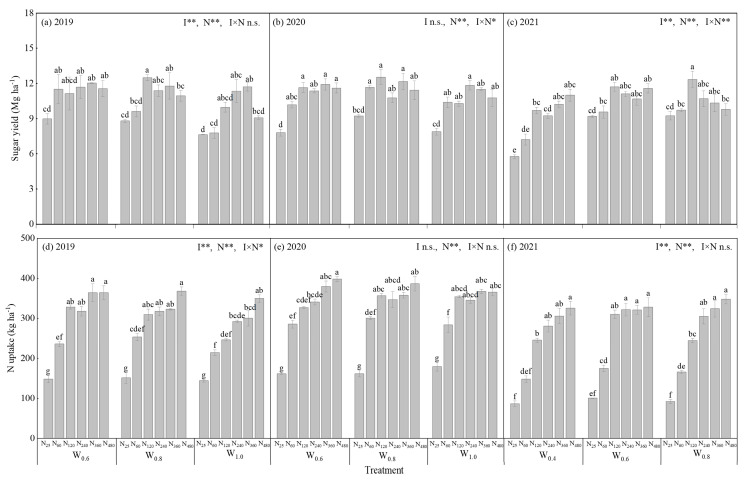
Sugar yield and nitrogen (N) uptake in different irrigation and nitrogen treatments in 2019, 2020, and 2021. (**a**) Sugar yield in 2019; (**b**) Sugar yield in 2020; (**c**) Sugar yield in 2021; (**d**) N uptake in 2019; (**e**) N uptake in 2020; and (**f**) N uptake in 2021. Bars are the means ± one standard error of the mean (*n* = 3). * and ** mean a significant difference at *p* < 0.05 and *p* < 0.01, respectively; n.s. means no significant difference (*p* > 0.05). Different letters indicate the significant difference between treatments at 0.05 level by Tukey’s HSD test in the same year. *W*_0.4_, *W*_0.6_, *W*_0.8,_ and *W*_1.0_ are irrigation amounts of 0.4, 0.6, 0.8, and 1.0 *ET_c_*, respectively. N_25_, N_60_, N_120_, N_240_, N_360,_ and N_480_ are nitrogen rates of 25, 60, 120, 240, 360, and 480 kg N ha^−1^, respectively.

The irrigation–nitrogen (I × N) interaction had no significant effect on sugar yield in 2019 (*p* > 0.05), but it had significant effects on sugar yield in 2020 and 2021 (*p* < 0.05). For the three years, the sugar yield varied from 7.6 to 12.5 Mg ha^−1^ in 2019, from 7.8 to 12.5 Mg ha^−1^ in 2020, and from 5.8 to 12.3 Mg ha^−1^ in 2021, respectively. The sugar yield increased and then generally stabilized with the increasing N rate under the same irrigation amount in the same year (Figure 1a–c).

#### 3.1.2. N Uptake

The I × N interaction had a significant effect on N uptake in 2019 (*p* < 0.05), but it had no significant effect on N uptake in 2020 and 2021 (*p* > 0.05). For the three years, the total N uptake varied from 145.5 to 364.2 kg ha^−1^ in 2019, from 161.8 to 398.8 kg ha^−1^ in 2020, and from 86.2 to 347.8 Mg ha^−1^ in 2021, respectively. In general, the N uptake increased with the increasing N rate under the same irrigation amount. However, most treatments showed little difference in N uptake under the same N rate in the same year (Figure 1d–f).

#### 3.1.3. NU_P_E, NU_t_E and NUE

**Figure 2:**
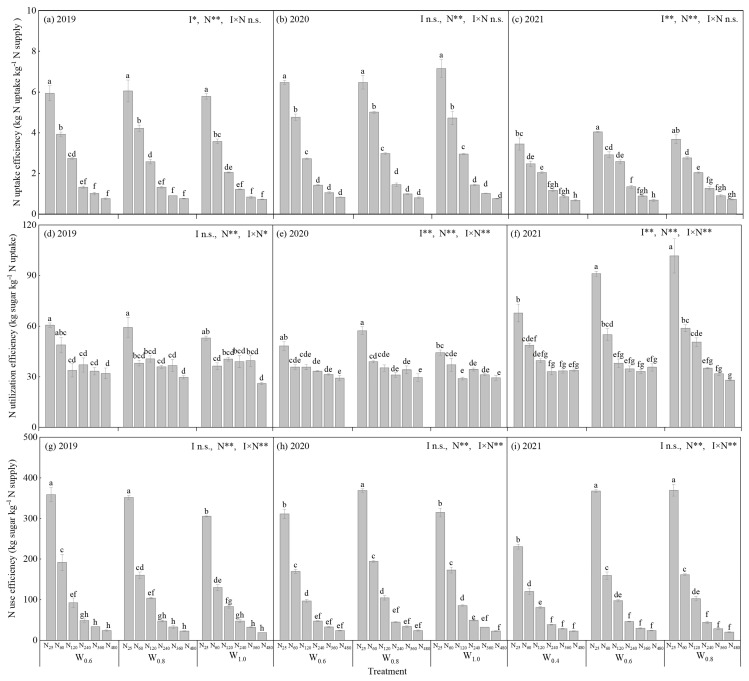
Nitrogen (N) uptake efficiency, utilization efficiency, and use efficiency of sugar beet in different irrigation and nitrogen treatments in 2019, 2020, and 2021. Bars are the means ± one standard error of the mean (*n* = 3). (**a**) N uptake efficiency in 2019; (**b**) N uptake efficiency in 2020; (**c**) N uptake efficiency in 2021; (**d**) N utilization efficiency in 2019; (**e**) N utilization efficiency in 2020; (**f**) N utilization efficiency in 2021; (**g**) N use efficiency in 2019; (**h**) N use efficiency in 2020; and (**i**) N use efficiency in 2021. * and ** mean a significant difference at *p* < 0.05 and *p* < 0.01, respectively; n.s. means no significant difference (*p* > 0.05). Different letters indicate the significant differences in the treatments at 0.05 level by Tukey’s HSD test in the same year. *W*_0.4_, *W*_0.6_, *W*_0.8,_ and *W*_1.0_ are irrigation amounts of 0.4, 0.6, 0.8, and 1.0 *ET_c_*, respectively. N_25_, N_60_, N_120_, N_240_, N_360,_ and N_480_ are nitrogen rates of 25, 60, 120, 240, 360, and 480 kg N ha^−1^, respectively.

The I × N interaction had no significant effect on NU_P_E in the three years (*p* > 0.05), but it had a significant effect on NU_t_E and NUE in the three years (*p* < 0.05). For the three years, the NU_P_E varied from 0.7 to 6.1 kg kg^−1^ in 2019, from 0.8 to 7.2 kg kg^−1^ in 2020, and from 0.7 to 4.0 kg kg^−1^ in 2021, respectively. The NU_t_E varied from 26.0 to 60.6 kg kg^−1^ in 2019, from 29.2 to 57.3 kg kg^−1^ in 2020, and from 28.0 to 101.7 kg kg^−1^ in 2021, respectively. The NUE varied from 18.9 to 359.0 kg kg^−1^ in 2019, from 22.4 to 369.1 kg kg^−1^ in 2020, and from 20.3 to 369.7 kg kg^−1^ in 2021, respectively. The trends of NU_P_E, NU_t_E, and NUE are roughly the same among the treatments, that is, the maxima of NU_P_E, NU_t_E, and NUE were observed at N_25_, and the minima of NU_P_E, NU_t_E, and NUE were observed at N_480_ for a given irrigation amount (Figure 2).

#### 3.1.4. Soil NO_3_-N Distribution in the 0–80 cm Soil Profile at Harvest

**Figure 3:**
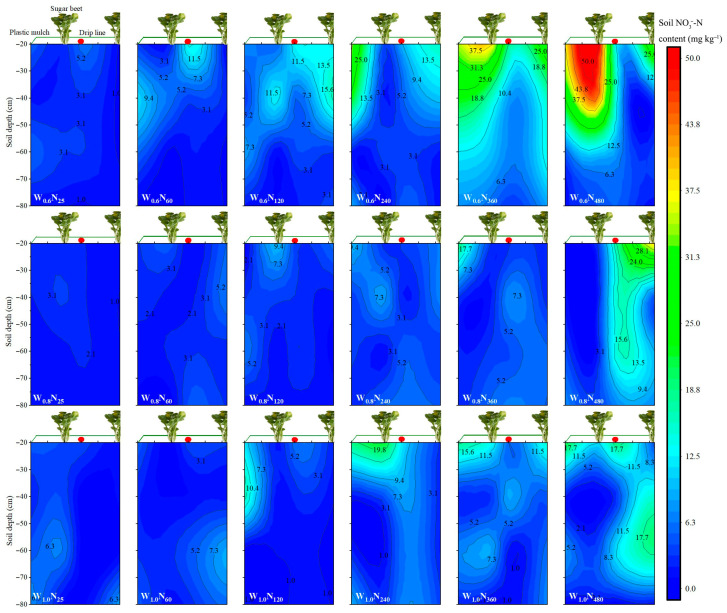
Examples of the spatial distribution of soil NO_3_^−^-N in each treatment after the experiment in 2019. W_0.6_, W_0.8,_ and W_1.0_ are irrigation amounts of 0.6, 0.8, and 1.0 *ET_c_*, respectively. N_25_, N_60_, N_120_, N_240_, N_360,_ and N_480_ are nitrogen rates of 25, 60, 120, 240, 360, and 480 kg N ha^−1^, respectively. The spatial distribution of soil NO_3_^−^-N in each treatment after the experiment in 2020 and 2021 can be found in Figure S7.

There was no significant difference in soil NO_3_-N contents among N_25_, N_60,_ and N_120_ under the same irrigation amount, and their soil NO_3_-N contents were lower. In general, soil NO_3_-N contents increased as N rates increased, and this phenomenon was more obvious with the higher N rates. By calculating the average soil NO_3_-N residual amount in the 0–80 cm soil layer of each irrigation amount during the three years, we found that the maximum average soil NO_3_-N accumulation was obtained at the W_0.4_, and the minimum was obtained at the W_1.0_. In the horizontal distance, with the drip line as the centre, soil NO_3_-N moved to both sides with water. Many treatments had a soil NO_3_-N accumulation zone, which was located on the soil surface layer or at a depth of 40 cm below the soil surface. This phenomenon became more obvious with the increase in the N rate. In addition, the soil NO_3_-N of W_0.8_N_480_ and W_1.0_N_480_ in 2019 and W_1.0_N_480_ in 2020 moved into deep soil layers, but that of other treatments was mainly distributed in the 0–60 cm soil layer in the three years (Figure 3; Figure S7).

### 3.2. N_c_ Dilution Curve Model

#### 3.2.1. Determination of Nc Dilution Curve Model

As shown in Figures S4 and S5, we found that increasing the N rate increased the dry matter and N concentration of sugar beet. In addition, the dry matter of sugar beet increased with the increase in days after planting, and the N concentration decreased as the days after planting increased.

**Figure 4:**
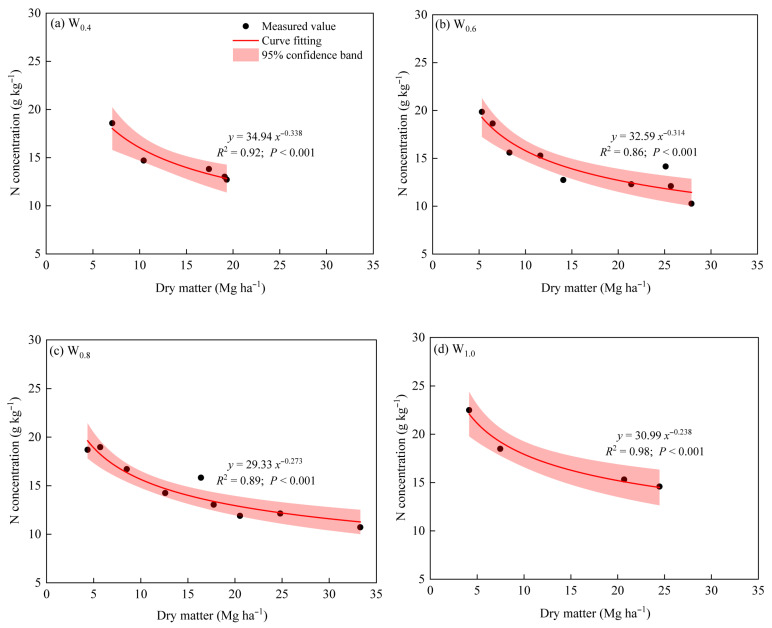
Critical nitrogen dilution curves for sugar beet under different irrigation amounts. (**a**) critical nitrogen dilution curves under W_0.4_ irrigation amount; (**b**) critical nitrogen dilution curves under *W*_0.6_ irrigation amount; (**c**) critical nitrogen dilution curves under *W*_0.8_ irrigation amount; and (**d**) critical nitrogen dilution curves under *W*_1.0_ irrigation amount. *W*_0.4_, *W*_0.6_, *W*_0.8_, and *W*_1.0_ mean irrigation amounts of 0.4, 0.6, 0.8, and 1.0 *ET_c_*, respectively.

Before selecting data points, one-way ANOVA was carried out on the dry matter of sugar beet of different N rates under each irrigation amount in the three years. Data points from each sampling from 78 to 160 days after planting in 2020 and from 81 to 162 days after planting in 2021 were used to determine the *N_c_* concentration points by following the method proposed by [28]. The *N_c_* dilution curve models with different irrigation amounts all showed good performance in the calibration dataset (*p* < 0.001, Figure 4). In addition, we found that model parameters *a* and *b* had significant quadratic correlations with the irrigation coefficients. Therefore, *a* and *b* were fitted linearly with the corresponding irrigation coefficients. The fitting equations were as follows:

(12) $$a=25.06\times I^{2}-\;42.64\times I+48.28,R^{2}=0.90,p<0.001$$

(13) $$b=-0.07\times I^{2}-0.07\times I+0.38,R^{2}=0.99,p<0.001$$

where I is the irrigation coefficient. Therefore, the *N*_c_ dilution curves of sugar beet under different irrigation amounts can be expressed as follows:

(14) $$N_{c}=(25.06\times I^{2}-\;42.64\times I+48.28)\times {DM}^{-(-0.07\times I^{2}-0.07\times I+0.38)},R^{2}=0.91,p<0.001$$

#### 3.2.2. Validation of N_c_ Dilution Curve Model

**Figure 5:**
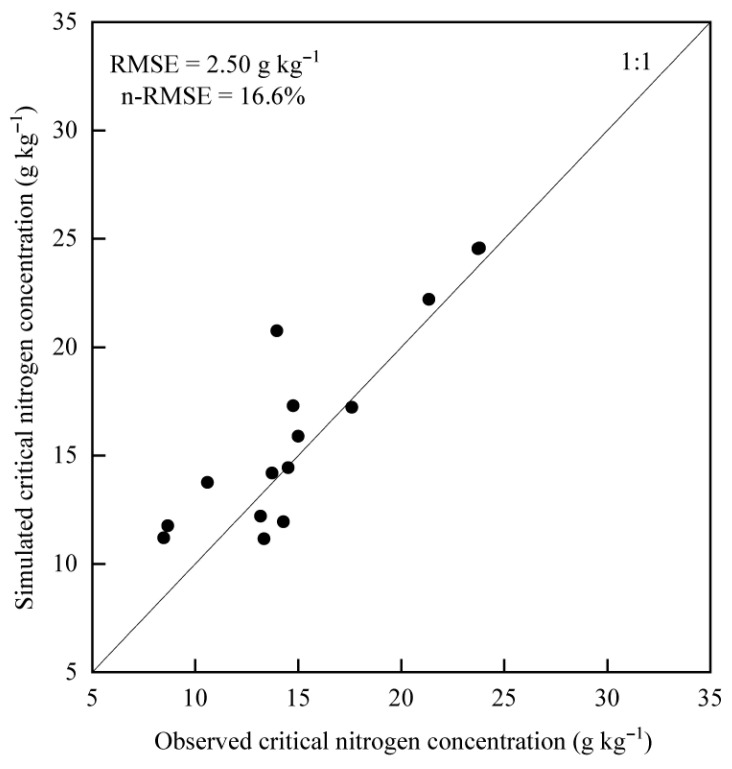
Comparison of simulated and observed critical nitrogen concentrations. Each black dot represents a paired data point of observed versus simulated critical nitrogen concentration.

The validation of the model was carried out using the data from 2019 under different irrigation levels (*W*_0.6_, *W*_0.8,_ and *W*_1.0_). As shown in Figure 5, the model accurately estimated the *N_c_* concentration with an RMSE of 2.50 g kg^−1^ and n-RMSE of 16.6%. The n-RMSE value was more than 10% but less than 20%, indicating that the established model was stable and could be further used for the diagnosis of N nutrition in the sugar beet growth period.

#### 3.2.3. N Nutrition Diagnosis Based on the Nc Dilution Curve Model

As shown in Figure S6, sugar beet is more prone to N deficit at the early growth stage (*NNI* < 1). In general, *NNI* showed an increasing trend with the increase in the N rate. For a given irrigation amount, *NNI* under N_25_ and N_60_ were all less than 1. At harvest, the *NNI* of most treatments were less than 1.

#### 3.2.4. Relationship Between Relative Sugar Yield and NNI

**Figure 6:**
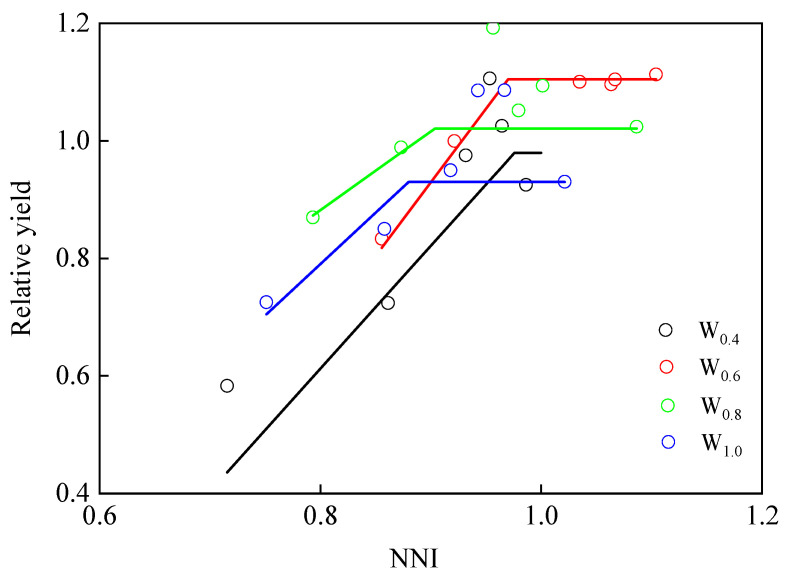
Relationship between relative sugar yield and nitrogen nutrition index (*NNI*) for four irrigation amounts and six nitrogen rates. The *NNI* data were averaged for N_25_, N_60_, N_120_, N_240_, N_360,_ and N_480_ treatments over three years. Colored lines represent different irrigation levels: black for *W*_0.4_, red for *W*_0.6_, green for *W*_0.8_, and blue for *W*_1.0_.

The relationships between relative sugar yield (*RY*) and *NNI* were expressed by linear-plateau function in different irrigation amounts (Figure 6). The regression models were:

(15) $$W_{0.4}:\;RY=\left\{\begin{array}{c} -1.06+2.09\;NNI,\;NNI<0.98\\ 0.98,\;NNI\geq 0.98\; \end{array}\right.\;R^{2}=\;0.82,\;p\;<\;0.01$$

(16) $$W_{0.6}:\;RY=\left\{\begin{array}{c} -1.33+2.51\;NNI,\;NNI<0.97\\ 1.10,\;NNI\geq 0.97\; \end{array}\right.\;R^{2}=\;0.99,\;\;p<\;0.01$$

(17) $$W_{0.8}:\;RY=\left\{\begin{array}{c} -0.19+1.34\;NNI,\;NNI<0.90\\ 1.02,\;NNI\geq 0.90\; \end{array}\right.\;R^{2}=\;0.70,\;p=\;0.01$$

(18) $$W_{1.0}:\;RY=\left\{\begin{array}{c} -0.61+1.75\;NNI,\;NNI<0.88\\ 0.93,\;NNI\geq 0.88\; \end{array}\right.\;R^{2}=\;0.93,\;p\;<\;0.01$$

Based on the relationships, for *NNI* = 0.97 of *W*_0.6_, the *RY* was highest, while for *NNI* > 0.97 of *W*_0.6_, the *RY* was generally stable at 1.10.

## 4. Discussion

### 4.1. Effects of Irrigation Amount and N Rate on Sugar Yield, N Uptake and Use Efficiency, and Soil NO_3_-N Residue

#### 4.1.1. Sugar Yield

The significant irrigation–nitrogen interaction on sugar yield of sugar beet observed in 2020 and 2021 was primarily driven by irrigation amount under N_25_ and N_60_, as well as variable responses to increasing N from 25 to 120 kg ha^−1^. For a given irrigation amount, we found that sugar yield increased and then roughly stabilized as the N rate increased. This aligns with findings from Kiymaz and Ertek [12], who reported optimal sugar yield at specific irrigation and N rates. The decline or stabilization in yield at higher N rates may be attributed to reduced sucrose content and increased impurities (Na, K, and amino-N) [12]. Similarly, reduced irrigation led to lower sugar yield due to more water accumulation in the taproot, which decreases sucrose content [21,49,50]. Therefore, controlled irrigation amount and N rate for increasing sugar yield are of vital importance.

#### 4.1.2. N Uptake and Use Efficiency

Analyzing crops’ N uptake and use efficiency can help to evaluate whether the N rate is suitable [9]. In this study, total N uptake was higher than observed in other studies [9,42,43], likely due to a combination of increased N supply, crop nutrient demand, and irrigation method. Some studies reported that plant N uptake was determined by both the plant N rate and N demand [43,51]. Thus, the treatment with higher N uptake can be explained by a higher growth rate and N rate (Figure S4). In addition, except for the different experimental weather conditions and N rates among the studies, another possible reason is that we used drip irrigation technology in our study. This technology has been demonstrated to improve plant nutrient uptake and yield [52], and it is widely used for saving water and fertilizer resources in agricultural production in arid and semi-arid regions.

We found that plant total N uptake showed opposite trends to NU_P_E, NU_t_E, and NUE with the increasing N rate. This inverse relationship is consistent with Armstrong et al. [51], who observed that sugar beet can exhaust the N from the soil during the growth period. Thus, the sugar beet under a lower N supply further exhausted the N in the soil and increased NU_P_E. The lower soil NO_3_-N residue content under the lower N supply treatment supports this explanation. The NU_P_E, NU_t_E, and NUE had a similar trend as observed for sugar beet under varying N rates under the same irrigation amounts. The high NU_t_E indicated effective remobilization of N under the lower N supply [53]. However, a lower N supply did not increase the sugar yield in the three years. In terms of pursuing a high sugar yield, this N rate (e.g., N_25_) is not desirable.

#### 4.1.3. Soil NO_3_-N Residue

In this study, soil NO_3_-N content and distribution were significantly affected by irrigation amount, N rate, and crop N uptake was also reported by Gorska et al. [54]. Excessive N input not only decreased N uptake and utilization efficiency but also increased the soil NO_3_-N residue, thereby increasing the risk of environmental pollution [55,56]. The irrigation–nitrogen interaction notably influenced the distribution of soil NO_3_-N. Increasing irrigation amount may decrease the soil NO_3_-N content in the 0–80 cm soil layer, likely due to enhanced nitrate leaching or deeper movement. These results concur with Badr et al. [57], who found that the soil NO_3_-N was readily soluble in water and moved with the water. Additionally, the root system of crops causes more water to move in the horizontal direction after entering the soil and accumulates on the edge of the wetting front. From a mechanistic perspective, drip fertigation improves NUE by synchronizing N supply with crop demand and reducing nitrate buildup beyond the root zone. Therefore, we should choose appropriate water and N management to meet the growing needs of crops, reduce waste of water and fertilizer resources, and ultimately achieve high yields.

### 4.2. N_c_ Dilution Curve Model

#### 4.2.1. Nc Dilution Curve Model Based on Dry Matter

In the current study, the selected data points of dry matter used to establish the *N*_c_ dilution curve were > 1 Mg ha^−1^ [28]. Despite this, the selected data points had a shortcoming in that most of the data points were collected when the dry matter yield was more > 2 Mg ha^−1^. Further studies should consider some data points of dry matter above but close to 1 Mg ha^−1^, to better verify the acceptability of the *N*_c_ dilution curve.

In agricultural practice, the *N*_c_ dilution curve is an important diagnostic tool to assess the N profit and loss status of crops at the growth stage; as long as the dry matter of crops is available, the N nutrition status of crops can be determined according to the *N*_c_ dilution curve [58,59]. For example, if the measured N concentration is on the *N*_c_ dilution curve, it indicates that the N application rate was optimal. On the contrary, if the measured N concentration is higher (or lower) than the *N*_c_ dilution curve, it indicates that there is an N surplus (or deficiency) in the plant [29,60]. However, there were some differences in the model parameters between our study and the previous studies. Parameter comparisons revealed noticeable differences between our model and previous studies. Firstly, sugar beet is a C3 crop, but we found that parameter *a* in the present study was lower than that of Greenwood et al. [31], who established a general *N*_c_ dilution curve for C3 crops (*a* = 57.0 g kg^−1^). Indeed, previous studies have shown that the parameters of the *N*_c_ dilution curve are affected by many factors, including agronomic practices, the ecological climate of crop growth, crop types, and varieties [37,38,58]. For example, the parameters *a* (43.6 g kg^−1^) of the *N*_c_ dilution curve of winter wheat in the Guanzhong Plain in China [29] were lower than the parameters *a* (53.5 g kg^−1^) of the *N*_c_ dilution curve of winter wheat in France [28]. Potato and tomato belong to the C3 group of crops, but the parameters *a* are not the same between potato (*a* = 53.0 g kg^−1^) and tomato (*a* = 45.3 g kg^−1^) [37,38]. It is indicated that *N*_c_ dilution curve parameters vary with crop species, cultivars, management practices, and climate conditions. Therefore, it is expected that each crop needs its model parameters [32]. Secondly, the value of parameters *b* in this study was different from that of other studies, and one of the main reasons was that the crop types were different [28,37,38,58]. For example, Qiang et al. [29] reported that the parameter *b* was higher in C3 species than in C4 species when the *N*_c_ dilution curve was based on dry matter. In addition, the parameters *b* indicate the decline in plant N concentration with crop growth and depend on plant N uptake relative to dry matter increase. This reflects physiological differences in nitrogen uptake and biomass allocation between crop types. Therefore, the decrease in plant N concentration during vegetative growth can be ascribed to the decreased N concentration per unit of dry matter.

#### 4.2.2. Optimal Irrigation Amount and N Rate Based on the Diagnosis of N Nutrition Status

In this study, the main purpose of establishing the *N*_c_ dilution curve was to diagnose the N nutritional status of crops by agronomic research methods, which have been applied widely in agricultural production [34,58]. Thus, real-time diagnosis of plant N nutrition status based on NNI is the basis for rational N application, promoting crop growth and increasing economic yield, and NNI is a reliable diagnostic tool for evaluating the N status of crops [26,48].

This study analyzed the variation range of NNI during the growth period of sugar beet under different water and N supplies and obtained the appropriate N application rate under different irrigation conditions for three years. We found that sugar beet was more prone to N deficit at the early stage of growth. Meanwhile, it was reported that maintaining soil N deficiency for 42 days before harvest improves sugar beet quality [61]. To improve the N use efficiency, absorption, and utilization, N fertilizer should be applied at the early growth stage of sugar beet, and there should be no excess N in the soil 4–6 weeks before harvest [22].

We found that the relationships between relative sugar yield (RY) and NNI were expressed by linear-plateau functions and accounted for more than 70% of the variation. A similar relationship was also found between RY and NNI in potatoes [37], maize [62], and greenhouse cherry tomato [63]. The relationships confirmed the link between N uptake and sugar yield of sugar beet, confirming the importance of NNI for predicting actual sugar yield with respect to potential sugar yield. In addition, for an NNI > 0.97 of W_0.6_, the RY was generally stable at 1.10 and higher than that of other irrigation amounts, which indicated that the optimal NNI was 0.97 under W_0.6_. Meanwhile, since the agricultural water resources of each sugar beet planting area are different, it is important to choose the appropriate N rate that corresponds to the actual irrigation amount and reduce the waste of water and N fertilizer resources.

## 5. Conclusions

This study demonstrated that optimizing irrigation and nitrogen (N) input significantly improved sugar beet yield and nitrogen use efficiency (NUE) under arid and semi-arid conditions. The maximum relative sugar yield (RY = 1.10) was achieved at an N nutrition index of 0.97 under 0.6 ETc irrigation, indicating that moderate irrigation coupled with proper N supply is optimal for both productivity and environmental protection. Although plant N uptake and concentration increased with N rate, higher NUE, NU_P_E, and NU_t_E were observed under lower N inputs. Excessive N application (480 kg ha^−1^) resulted in NO_3_^−^–N accumulation below 60 cm soil depth, implying increased leaching risk. A crop-specific Nc dilution curve based on dry matter was developed with a stable fit (n-RMSE = 16.6%), and its parameters showed significant quadratic responses to irrigation level. Compared to curves established for other C_3_ crops and regions, the differences in parameter values highlighted the need for regionally adapted Nc models. Overall, the Nc curve approach proves effective for diagnosing sugar beet N status and provides a valuable tool for improving N management in similar agro-ecological zones.

## Data Availability

Data are contained within the article and Supplementary Materials.

## Supplementary Materials

The following supporting information can be downloaded at: https://www.mdpi.com/article/10.3390/plants14132055/s1, Figure S1: (a) Daily average air temperature, (b) precipitation, (c) solar radiation and (d) reference crops evapotranspiration (ET_0_) during growing seasons of sugar beet in 2019, 2020, and 2021; Figure S2: Layout of sugar beet drip irrigation under plastic mulch and soil sampling locations; Figure S3: Timing and amounts of drip irrigation under plastic mulching and the proportions of total fertilizer applied each time for sugar beet in (a) 2019, (b) 2020 and (c) 2021. Figure S4: The changes of aboveground biomass over time under different treatments in 2019, 2020 and 2021. Different letters indicate the significance on the same day after planting between different treatments at 0.05 level by Tukey’s HSD test. W_0.4_, W_0.6_, W_0.8_ and W_1.0_ are irrigation amounts of 0.4, 0.6, 0.8 and 1.0 *ET_c_*, respectively. N_25_, N_60_, N_120_, N_240_, N_360_ and N_480_ are nitrogen rates of 25, 60, 120, 240, 360 and 480 kg N ha^-1^, respectively. The black labels in 2020 and 2021are the selected calibration dataset, and the black labels in 2019 are the selected validation dataset. Figure S5: The changes in nitrogen concentration of dry matter over time under different treatments in 2019, 2020 and 2021. Bars are the means ± one standard error of the mean (*n* = 3). W_0.4_, W_0.6_, W_0.8_ and W_1.0_ are irrigation amounts of 0.4, 0.6, 0.8 and 1.0 *ET_c_*, respectively. N_25_, N_60_, N_120_, N_240_, N_360_ and N_480_ are nitrogen rates of 25, 60, 120, 240, 360 and 480 kg N ha^-1^, respectively. Figure S6: The dynamics of nitrogen nutrition index (NNI) over time under different irrigation amounts. The blue dashed line represents the NNI = 1, which means that sugar beet is under optimal nitrogen nutrition status. W_0.4_, W_0.6_, W_0.8_ and W_1.0_ are irrigation amounts of 0.4, 0.6, 0.8 and 1.0 *ET_c_*, respectively. N_25_, N_60_, N_120_, N_240_, N_360_ and N_480_ are nitrogen rates of 25, 60, 120, 240, 360 and 480 kg N ha^-1^, respectively. Figure S7: Spatial distribution of Soil NO_3_^−^-N in each treatment after the experiment in 2020 (a) and 2021 (b). W_0.4_, W_0.6_, W_0.8_ and W_1.0_ are irrigation amounts of 0.4, 0.6, 0.8 and 1.0 *ET_c_*, respectively. N_25_, N_60_, N_120_, N_240_, N_360_ and N_480_ are nitrogen rates of 25, 60, 120, 240, 360 and 480 kg N ha^−1^, respectively.
