# Supplementary material for: Optimization of Irrigation Amount and Nitrogen Rate of Drip-Fertigated Sugar Beet Based on Sugar Yield, Nitrogen Use Efficiency, and Critical Nitrogen Dilution Curve in the Arid Southern Xinjiang of China
Source: Plants (Basel). 2025 Jul 4;14(13):2055. doi: 10.3390/plants14132055 (PMC12252462; doi:10.3390/plants14132055)
Supplement: Supplementary file 1 [file plants-14-02055-s001.zip › plants-3708508-supplementary.pdf]

Figures

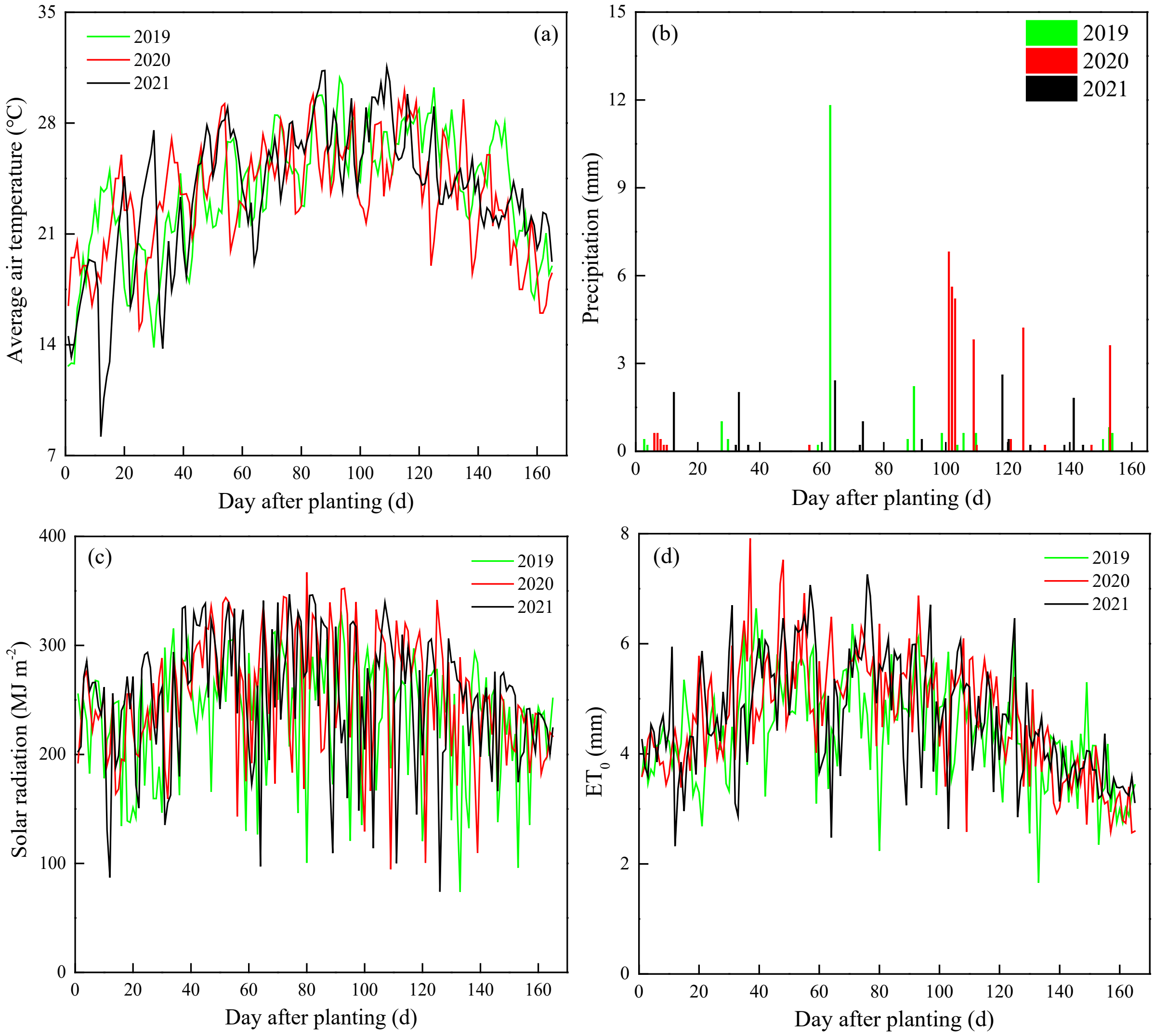

Figure S1. (a) Daily average air temperature, (b) precipitation, (c) solar radiation and (d) reference crops evapotranspiration (ET_0) during growing seasons of sugar beet in 2019, 2020, and 2021.

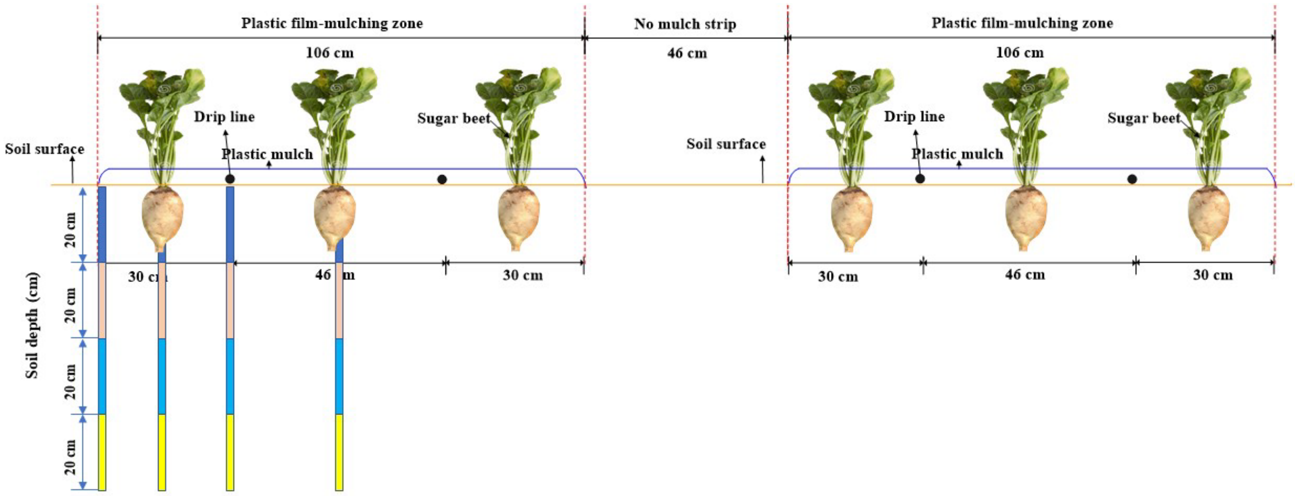

Figure S2. Layout of sugar beet drip irrigation under plastic mulch and soil sampling locations.

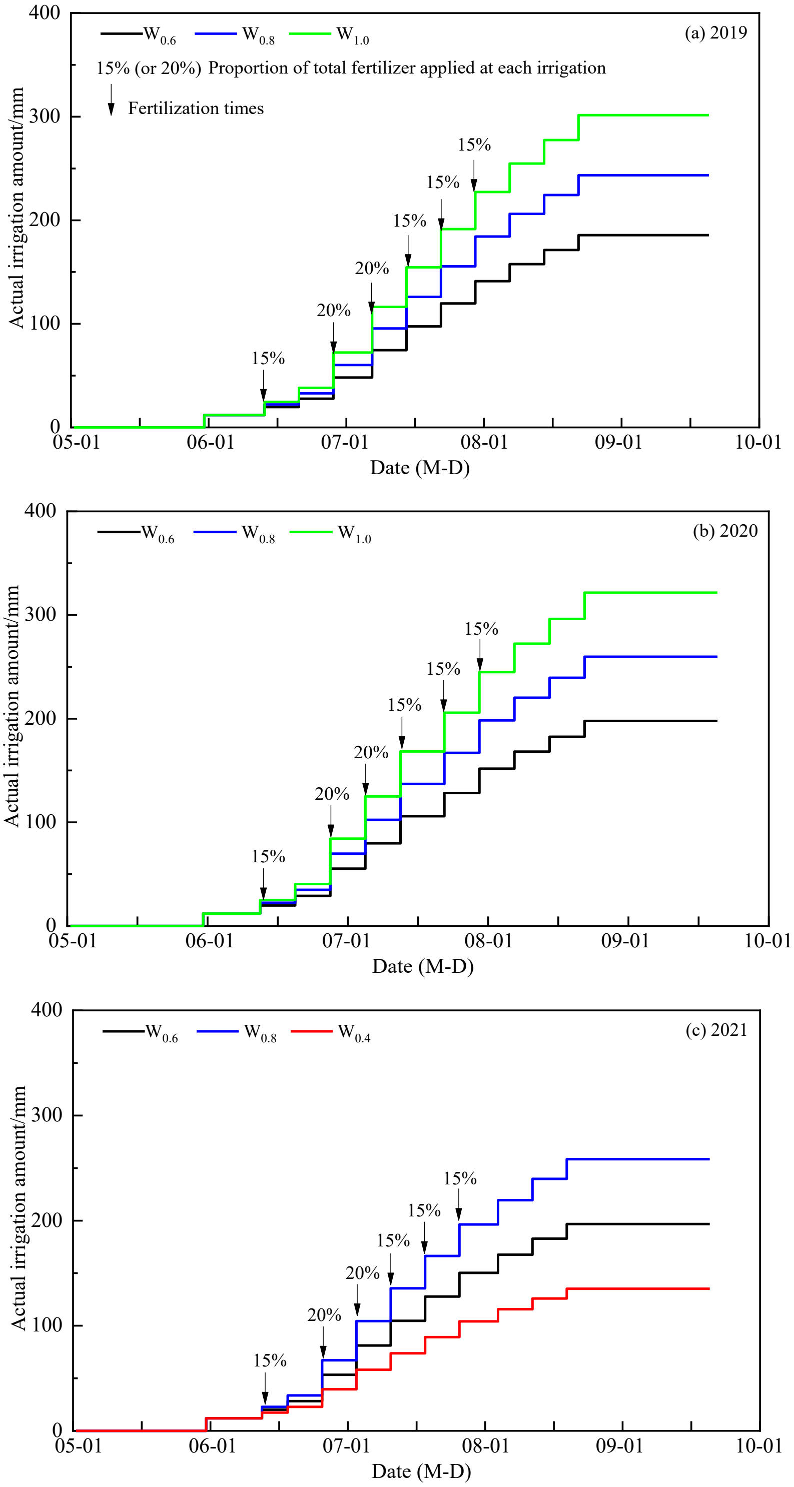

Figure S3. Timing and amounts of drip irrigation under plastic mulching and the proportions of total fertilizer applied each time for sugar beet in (a) 2019, (b) 2020 and (c) 2021.

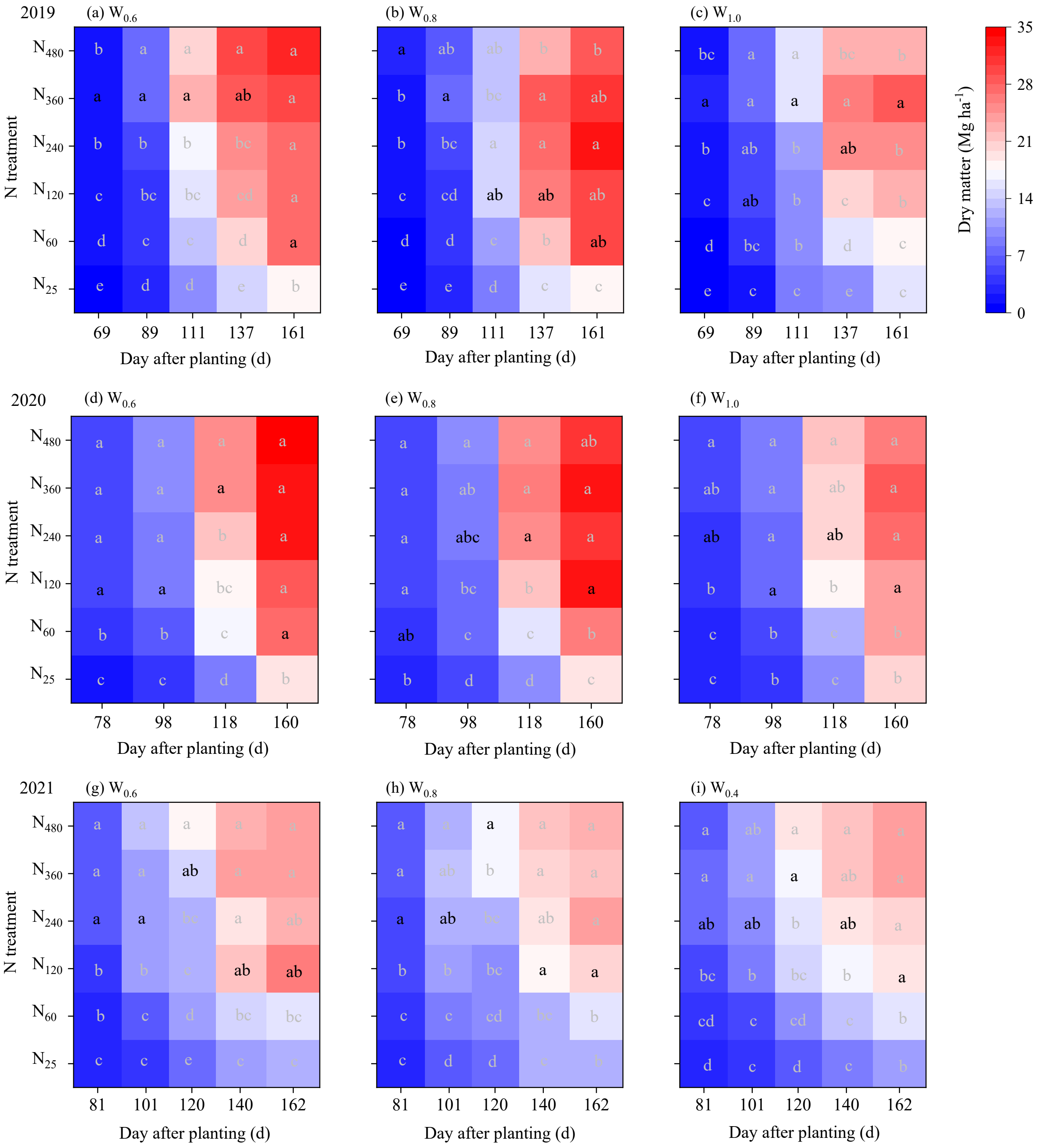

Figure S4. The changes of aboveground biomass over time under different treatments in 2019, 2020 and 2021. Different letters indicate the significance on the same day after planting between different treatments at 0.05 level by Tukey's HSD test. $W_{0.4}$, $W_{0.6}$, $W_{0.8}$ and $W_{1.0}$ are irrigation amounts of 0.4, 0.6, 0.8 and 1.0 ET_c, respectively. N_{25}, N_{60}, N_{120}, N_{240}, N_{360} and N_{480} are nitrogen rates of 25, 60, 120, 240, 360 and 480 kg N ha^{-1}, respectively. The black labels in 2020 and 2021are the selected calibration dataset, and the black labels in 2019 are the selected validation dataset.

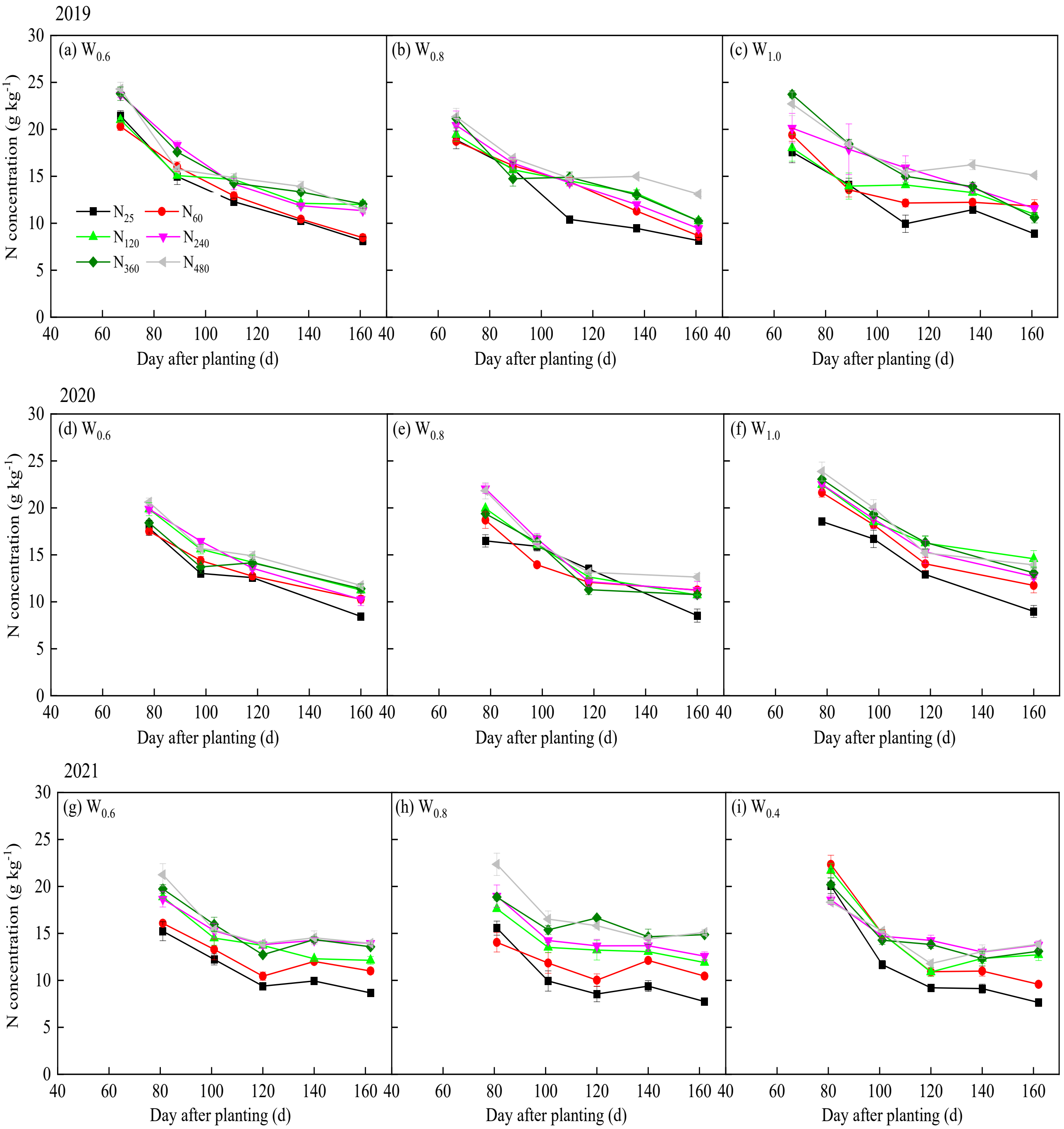

Figure S5. The changes in nitrogen concentration of dry matter over time under different treatments in 2019, 2020 and 2021. Bars are the means ± one standard error of the mean (n = 3). $W_{0.4}$, $W_{0.6}$, $W_{0.8}$ and $W_{1.0}$ are irrigation amounts of 0.4, 0.6, 0.8 and 1.0 ET_c, respectively. N_{25}, N_{60}, N_{120}, N_{240}, N_{360} and N_{480} are nitrogen rates of 25, 60, 120, 240, 360 and 480 kg N ha^{-1}, respectively.

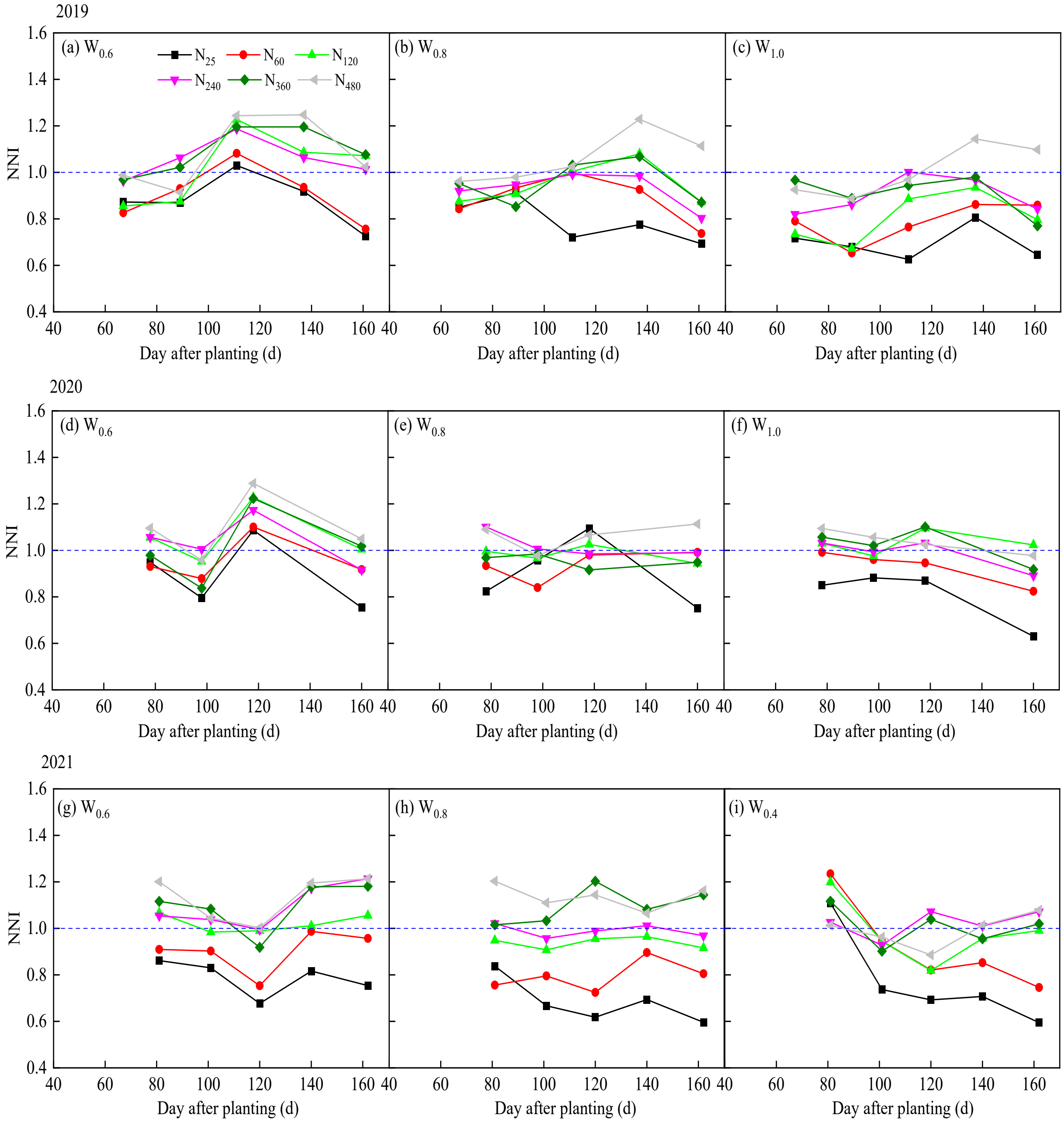

Figure S6. The dynamics of nitrogen nutrition index (NNI) over time under different irrigation amounts. The blue dashed line represents the NNI = 1, which means that sugar beet is under optimal nitrogen nutrition status. $W_{0.4}$, $W_{0.6}$, $W_{0.8}$ and $W_{1.0}$ are irrigation amounts of 0.4, 0.6, 0.8 and 1.0 ET_c, respectively. N_{25}, N_{60}, N_{120}, N_{240}, N_{360} and N_{480} are nitrogen rates of 25, 60, 120, 240, 360 and 480 kg N ha^{-1}, respectively.

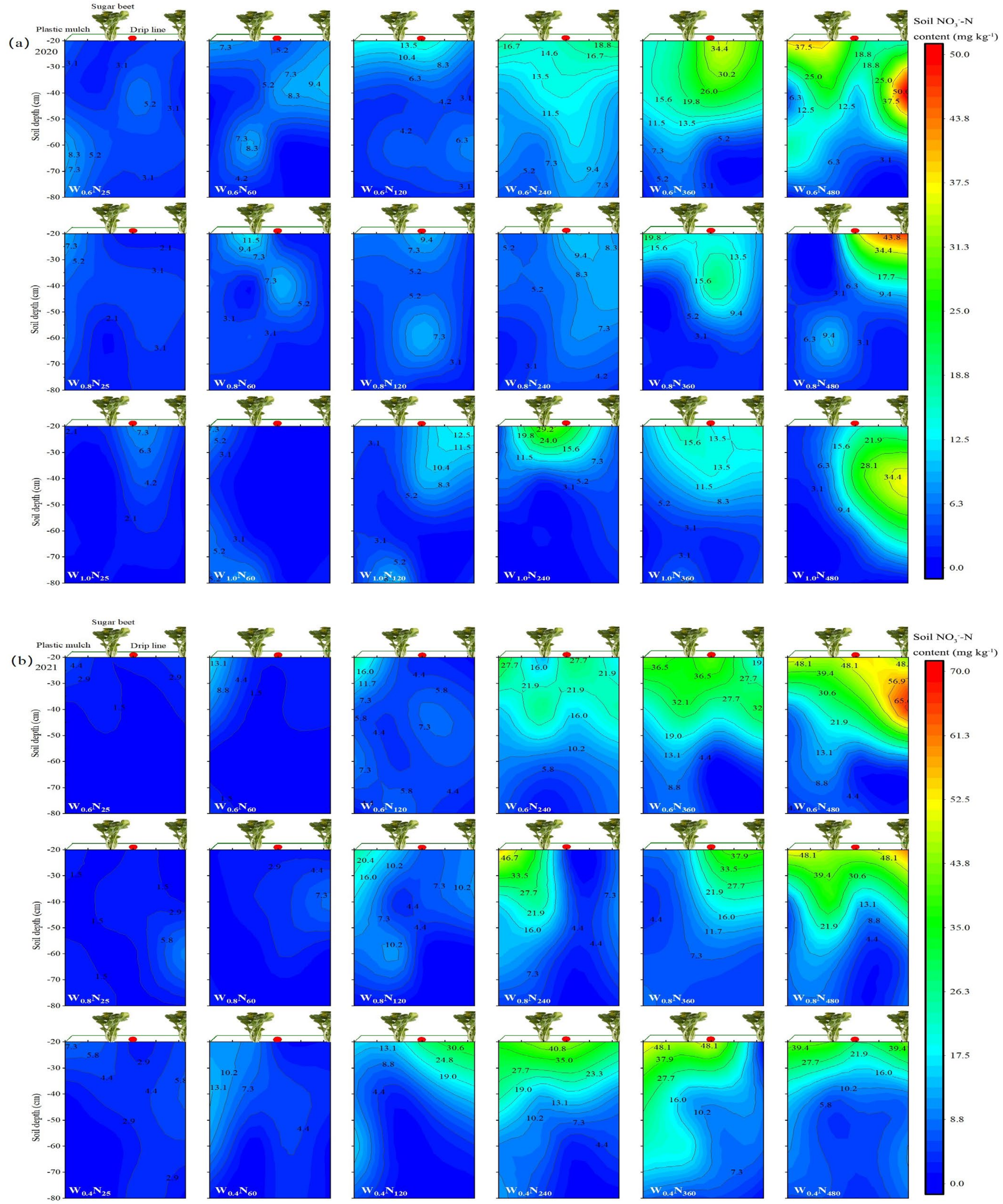

Figure S7. Spatial distribution of Soil NO_3^--N in each treatment after the experiment in 2020 (a) and 2021 (b). $W_{0.4}$, $W_{0.6}$, $W_{0.8}$ and $W_{1.0}$ are irrigation amounts of 0.4, 0.6, 0.8 and 1.0 ET_c, respectively. N_{25}, N_{60}, N_{120}, N_{240}, N_{360} and N_{480} are nitrogen rates of 25, 60, 120, 240, 360 and 480 kg N ha^{-1}, respectively.
